# Are there pulmonary sequelae in patients recovering from COVID-19?

**DOI:** 10.1186/s12931-020-01550-6

**Published:** 2020-10-30

**Authors:** Paola Rogliani, Luigino Calzetta, Angelo Coppola, Ermanno Puxeddu, Gianluigi Sergiacomi, Dejanira D’Amato, Antonio Orlacchio

**Affiliations:** 1 Unit of Respiratory Medicine, Department of Experimental Medicine, University of Rome “Tor Vergata”, Via Montpellier 1, 00133 Rome, Italy; 2 Division of Respiratory Medicine, University Hospital “Policlinico Tor Vergata”, Rome, Italy; 3 Department of Medicine and Surgery, Respiratory Disease and Lung Function Unit, University of Parma, Parma, Italy; 4 Department of Diagnostic Imaging, Molecular Imaging, Interventional Radiology and Radiotherapy, University of Rome Tor Vergata, Rome, Italy; 5 Department of Surgical Sciences, Unit of Radiology, University of Rome “Tor Vergata”, Rome, Italy; 6 Emergency Radiology Unit, University Hospital “Policlinico Tor Vergata”, Rome, Italy

**Keywords:** COVID-19, Pulmonary fibrosis, HRCT

## Abstract

It has been recently hypothesized that infection by severe acute respiratory syndrome coronavirus 2 (SARS-CoV-2) may lead to fibrotic sequelae in patients recovering from coronavirus disease 2019 (COVID-19). In this observational study, hospitalized patients with COVID-19 had a HRCT of the chest performed to detect the extension of fibrotic abnormalities via Hounsfield Units (HU). At follow-up, the lung density significantly improved in both lungs and in each lobe of all patients, being in the normal range (− 950 to − 700 HU). This study provides preliminary evidence that hospitalized patients with mild-to-moderate forms of COVID-19 are not at risk of developing pulmonary fibrosis.

## Introduction

Infection by severe acute respiratory syndrome coronavirus 2 (SARS-CoV-2) leading to coronavirus disease 2019 (COVID-19) is characterized by non-specific symptoms. The disease presentation may range from lack of clinical signs in asymptomatic patients to severe interstitial pneumonia, acute lung injury (ALI) and acute respiratory distress syndrome (ARDS) [1]. Moving from the evidence that infection by other respiratory coronaviruses is associated with significant post-viral lung fibrosis, it has been recently hypothesized that there could also be fibrotic consequences related with COVID-19 [1, 2].

In order to assess the real risk of developing post-COVID-19 pulmonary fibrosis, we have carried out an observational study in hospitalized COVID-19 patients with high-resolution computed tomography (HRCT) at hospital admission and follow-up.

## Materials and methods

### Study design

A high-quality, single-group, prospective, observational study was performed in agreement with the International Society for Pharmacoeconomics and Outcomes Research (ISPOR) recommendations at the Unit of Respiratory Disease at the University Hospital “Tor Vergata” in Rome (Italy) [3]. The study was approved by the local ethics committee of University Hospital “Policlinico Tor Vergata", Rome, Italy (protocol no. 83/20, 2020).

### Selection of patients

All the COVID-19 patients hospitalized at the ward of “Respiratory Medicine” between March 16th 2020 and April 16th 2020 who underwent HRCT of the chest both at admission and at follow-up were consecutively included in the study.

The severity of COVID-19 was ranked in agreement with arterial blood gas analysis findings, namely mild in the presence normal ratio of the partial pressure of oxygen to the inspired fraction of oxygen (P/F) or of mild hypoxemia, moderate for picture of ALI, and severe for picture of ARDS [4, 5].

### Endpoints

The primary endpoint of this study was the change from baseline in Hounsfield Units (HU), that has been shown to be an effective method to identify interstitial lung abnormalities [6].

The secondary endpoints were the change from baseline in Total Severity Score (TSS) [7] and the presence of significant extension of fibrotic abnormalities at follow-up [8]. HRCT features including lobar distribution and presence of ground glass opacities (GGO), consolidation, and linear opacities were analysed. Each lung lobe was eveluated for the percentage of involvement by assigning a score of 0 for 0% involvement, 1 for 1–25% involvement, 2 for 26–50% involvement, 3 for 51–75% involvement, and 4 for 76–100% involvement. The TSS, ranging from 0 to 20, was reached by summing the score of the lung lobes [7]. Extension of fibrotic abnormalities was assessed as previously described [8]. Two radiologists reviewed all images independently blinded to the clinical information.

Further variables were investigated at follow-up, namely the lung function via pulmonary function test to assess forced expiratory volume in 1st s (FEV_1_) and forced vital capacity (FVC), sub-maximal exercise test via six-minute walk test (6MWT), and dyspea via Borg scale.

The follow-up visits were performed between April 27th 2020 and May 29th 2020.

### Statistical analysis

The relationship between the change from baseline in HU and TSS was assessed via the Pearson correlation coefficient. Data were reported as mean and 95% Confidence Interval (95%CI) or median with range and interquartile range (IQR), with statistical significance set for *p* < 0.05. HU was assessed via the IntelliSpace software (Philips, Milan, Italy) and GraphPad Prism 5 (La Jolla, CA, USA) was used to perform the statistical analysis.

## Results

**Fig. 1:**
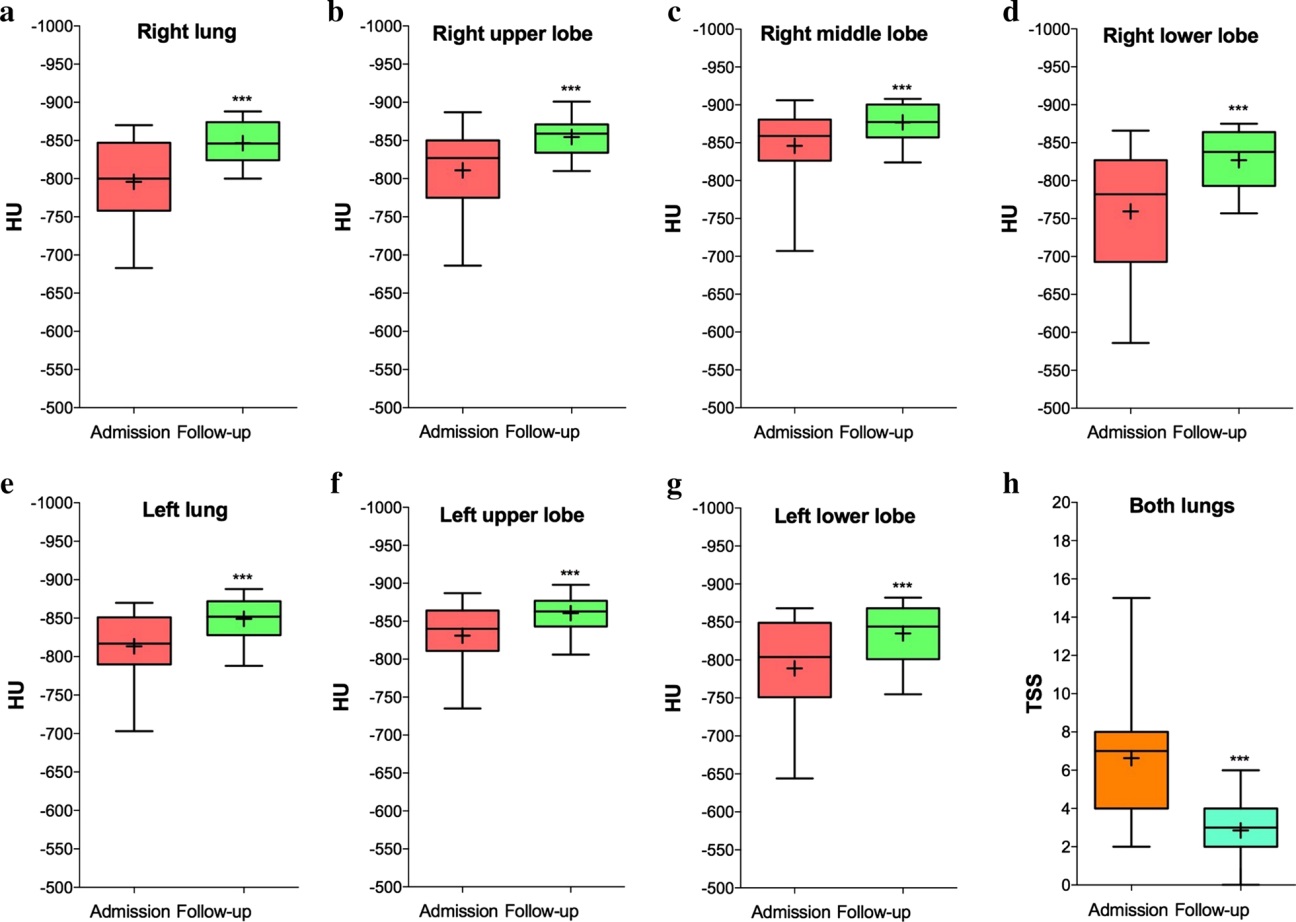
Analysis of HU in lungs (**a**, **e**) and lobes (**b**, **c**, **d**, **f**, **g**) and TSS (**h**) in COVID-19 patients (*N* = 27) at hospital admission and follow-up. ***p < 0.001 between groups (compared via paired *t*-test). The box and whisker plots show a 6-number data summary: minimum, 1st quartile, median, mean ( +), 3rd quartile, and maximum. The box is divided at the median. The length of the box is the interquartile range. The 1st quartile is the bottom line. The 3rd quartile is the top line. *COVID-19* coronavirus disease 2019, *HU* Hounsfield Units, *TSS* total severity score

Twenty-seven patients out of the 109 hospitalized subjects were included in this study as they had HRCT at both admission and follow-up. The patients baseline characteristics were: age 60.79 years (95%CI 57.11–64.46), 20 men and 7 women. Arterial blood gas analysis showed in 11.11% of patients a picture of ARDS, in 37.04% of ALI, 18.52% of mild hypoxemia, and 33.33% had a normal *P*/*F* ratio [5]. Patients were treated with hydroxychloroquine (100%), macrolides (96.30%), antiviral drugs (92.59%), low-molecular weight heparin (92.59%), corticosteroids (77.78%), tocilizumab (37.04%). 33.33% of patients received noninvasive ventilation, the remaining oxygen therapy as needed. No patients underwent invasive mechanical ventilation. Although the HU of both lungs was in the normal range (− 950 to − 700 HU) at hospital admission in most patients (HU: median − 810, IQR − 773 to − 838, range − 696 to − 870), GGO (HU cut-off: − 700) [6] was detected in nearly a quarter of patients at the level of the left and right lower lobes (HU: median − 792, IQR − 72 to − 829, range − 586 to − 868). At follow-up, 47.74 days (95%CI 43.42–52.06) post-admission, the lung density significantly (*p* < 0.001, assessed via paired *t*-test) improved in both the lungs (delta HU: − 45.16) and in each lobe, including the left and right lower lobes (delta HU: − 67.6), being in the normal range and indicating the lack of GGO (Fig. 1a–g) [6]. TSS also significantly (*p* < 0.001, assessed via paired *t*-test) improved at follow-up (TSS: median 7, IQR 5–8, range 2–15) when compared with admission (TSS: median 3, IQR: 2–4, range: 0–6) (Fig. 1h). In none of the follow-up HRCTs significant extension of fibrotic abnormalities including reticular opacities, traction bronchiectasis and honeycombing were detected.

**Fig. 2:**
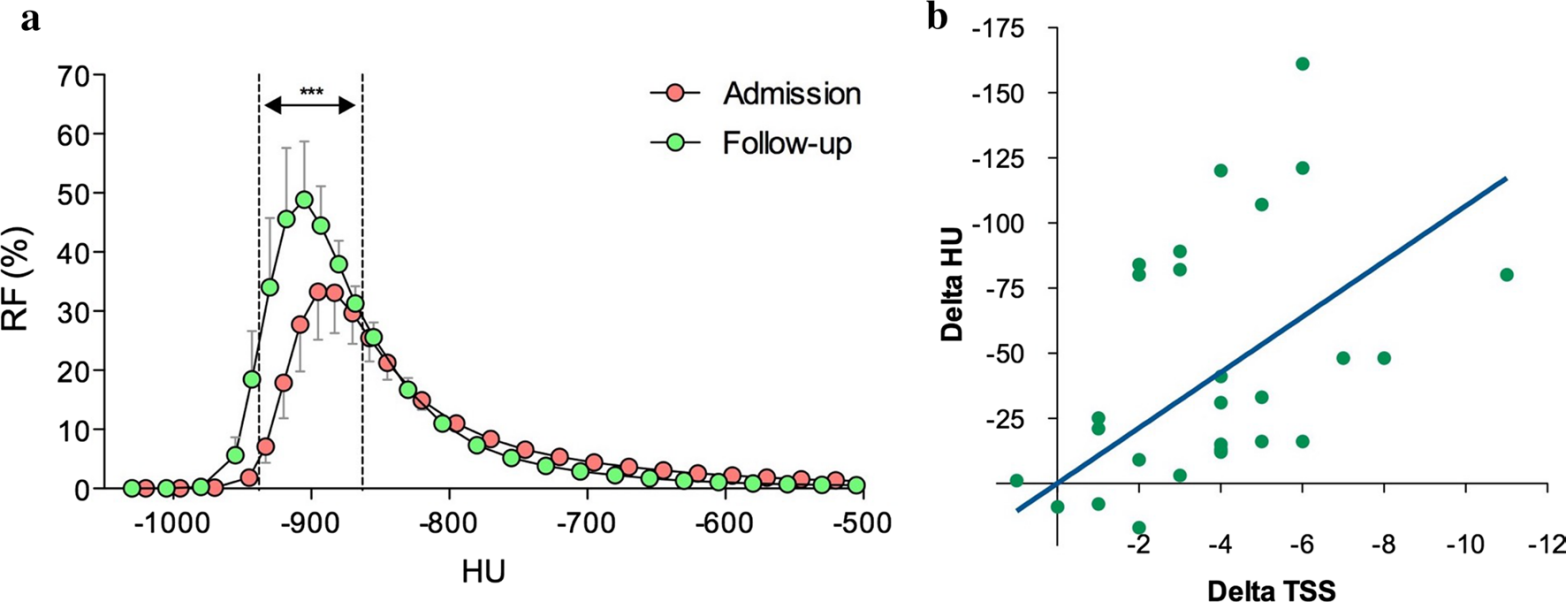
Analysis of density histogram curves (**a**) and correlation between HU and TSS (**b)** in COVID-19 patients (*N* = 27) at hospital admission and follow-up. ***p < 0.001 between groups (compared via two-way ANOVA). Data in (**a**) are reported as mean and 95%CI. *CI* confidence interval, *COVID-19* coronavirus disease 2019, *HU* Hounsfield Units, *RF* relative frequency, *TSS* total severity score

The changes from baseline in HU detected in both the lungs (density histogram curves with comparison reported in Fig. 2a) and TSS were plotted together (Fig. 2b), leading to a significant correlation (*p* < 0.05; slope 10.66, 95%CI 6.91–14.40; Pearson *r* 0.43, 95%CI 0.06–0.70). Pulmonary opacity recovery detected at follow-up was not statistically modulated either by disease severity and treatments (*p* > 0.05 via two-way analysis of variance) during hospitalization.

At follow-up, the percentage of predicted values of FEV_1_ (percentage predicted: 101.94%, 95%CI 91.23–112.66%), FVC (percentage predicted: 109.28%, 95%CI 97.52–121.03%), and the distance covered during the 6MWT (percentage predicted: 101.89%, 95%CI 93.89–109.88%) were in the normal range. The post-6MWT sensation of dyspnoea was clinically and statistically significant (delta pre-post 6MWT Borg scale: median 1.5, IQR 0.3–2, range 0–5; *p* < 0.05) [9].

## Discussion

This study provides the preliminary evidence that in hospitalized patients with prevalently mild-to-moderate forms of COVID-19 pulmonary opacity was completely recovered at follow-up, with no evidence of any fibrotic abnormality. Interestingly, at follow-up also lung function and exercise capacity were in the normal range. These findings suggest that these patients are not at risk of developing post-COVID-19 pulmonary fibrosis. However, also considering that abnormal pulmonary function was detected in COVID-19 patients at time of hospital discharge [10], the results of this study have to be confirmed in larger and longer studies, and verified also in patients with severe COVID-19.

Indeed, the main limit of this study is related with the preliminary nature of the investigation, as the analysis was performed on a small sample size. However, we have to highlight that no restrictions in the inclusion of patients were applied, and that all the patients were chosen consecutively at hospital admission, regardless of age and sex. This last point is important to prevent potential bias, as older male patients show more severe COVID-19 symptoms when compared to younger female patients [11].

In any case, despite the intrinsic limitations of this study, here we provided for the first time the suggestion that HU and TSS are directly correlated, at least in the COVID-19 population investigated in this study.

## Data Availability

The datasets used and/or analysed during the current study are available from the corresponding author on reasonable request.

## Abbreviations

ALI: Acute lung injury
ARDS: Acute respiratory distress syndrome
COVID-19: Coronavirus disease 2019
GGO: Ground glass opacities
HRCT: High-resolution computed tomography
HU: Hounsfield Units
IQR: Interquartile range
SARS-CoV-2: Severe acute respiratory syndrome coronavirus 2
TSS: Total Severity Score
6MWT: Six-minute walk test

## References

[1] Spagnolo P, Balestro E, Aliberti S, Cocconcelli E, Biondini D, Casa GD (2020). Pulmonary fibrosis secondary to COVID-19: a call to arms?. Lancet Respir Med.

[2] George PM, Wells AU, Jenkins RG (2020). Pulmonary fibrosis and COVID-19: the potential role for antifibrotic therapy. Lancet Respir Med.

[3] Berger ML, Dreyer N, Anderson F, Towse A, Sedrakyan A, Normand SL (2012). Prospective observational studies to assess comparative effectiveness: the ISPOR good research practices task force report. Value Health.

[4] Spagnolo P, Balestro E, Aliberti S, Cocconcelli E, Biondini D, Casa GD (2020). Pulmonary fibrosis secondary to COVID-19: a call to arms?. Lancet Respir Med.

[5] Karbing DS, Kjaergaard S, Smith BW, Espersen K, Allerod C, Andreassen S (2007). Variation in the PaO2/FiO2 ratio with FiO2: mathematical and experimental description, and clinical relevance. Crit Care.

[6] Ohkubo H, Kanemitsu Y, Uemura T, Takakuwa O, Takemura M, Maeno K (2016). Normal lung quantification in usual interstitial pneumonia pattern: the impact of threshold-based volumetric CT analysis for the staging of idiopathic pulmonary fibrosis. PLoS ONE.

[7] Li K, Fang Y, Li W, Pan C, Qin P, Zhong Y (2020). CT image visual quantitative evaluation and clinical classification of coronavirus disease (COVID-19). Eur Radiol.

[8] Mura M, Porretta MA, Bargagli E, Sergiacomi G, Zompatori M, Sverzellati N (2012). Predicting survival in newly diagnosed idiopathic pulmonary fibrosis: a 3-year prospective study. Eur Respir J.

[9] Ries AL (2005). Minimally clinically important difference for the UCSD shortness of breath questionnaire, Borg scale, and visual analog scale. COPD.

[10] Mo X, Jian W, Su Z, Chen M, Peng H, Peng P (2020). Abnormal pulmonary function in COVID-19 patients at time of hospital discharge. Eur Respir J.

[11] Borges do Nascimento IJ, von Groote TC, O’Mathúna DP, Abdulazeem HM, Henderson C, Jayarajah U (2020). Clinical, laboratory and radiological characteristics and outcomes of novel coronavirus (SARS-CoV-2) infection in humans: a systematic review and series of meta-analyses. PLoS ONE.

